# Cheminformatics Modeling of Gene Silencing for Both Natural and Chemically Modified siRNAs

**DOI:** 10.3390/molecules27196412

**Published:** 2022-09-28

**Authors:** Xialan Dong, Weifan Zheng

**Affiliations:** BRITE Institute and Department of Pharmaceutical Sciences, College of Health and Sciences (CHAS), North Carolina Central University, Durham, NC 27707, USA

**Keywords:** gene silencing, siRNA, chemically modified siRNA, BCUT descriptors, cheminformatics

## Abstract

In designing effective siRNAs for a specific mRNA target, it is critically important to have predictive models for the potency of siRNAs. None of the published methods characterized the chemical structures of individual nucleotides constituting a siRNA molecule; therefore, they cannot predict the potency of gene silencing by chemically modified siRNAs (cm-siRNA). We propose a new approach that can predict the potency of gene silencing by cm-siRNAs, which characterizes each nucleotide (NT) using 12 BCUT cheminformatics descriptors describing its charge distribution, hydrophobic and polar properties. Thus, a 21-NT siRNA molecule is described by 252 descriptors resulting from concatenating all the BCUT values of its composing nucleotides. Partial Least Square is employed to develop statistical models. The Huesken data (2431 natural siRNA molecules) were used to perform model building and evaluation for natural siRNAs. Our results were comparable with or superior to those from Huesken’s algorithm. The Bramsen dataset (48 cm-siRNAs) was used to build and test the models for cm-siRNAs. The predictive *r*^2^ of the resulting models reached 0.65 (or Pearson *r* values of 0.82). Thus, this new method can be used to successfully model gene silencing potency by both natural and chemically modified siRNA molecules.

## 1. Introduction

Specific gene silencing has shown great potential to elucidate gene function, identify drug targets, and develop more specific therapeutics than are currently available [1,2,3,4,5]. RNA interference (RNAi) is a post-transcriptional gene regulatory mechanism through which expression of a specific gene can be knocked down, which can be triggered either by short interfering RNAs (siRNAs) or by microRNAs (miRNAs) [6,7]. The miRNAs are endogenous noncoding RNAs; they usually bind target mRNAs with partial complementarity, mostly involving residues 1–8 (the seed region) [8]. Unlike siRNAs, which regulate mRNA levels through a cleavage event, miRNAs function by attenuating translation^8^. Here, we focus on siRNA-mediated gene silencing.

A siRNA is a double-stranded RNA molecule consisting of 21–23 nucleotides (21–23 NTs) with 2-NT overhangs at the 3′ ends and phosphate groups at 5′ ends [9,10]. siRNA molecules can be exogenously introduced or generated by RNase III-type enzyme Dicer from long dsRNA or hairpin RNA [6]. The two strands of a siRNA molecule have sequences that are sense and antisense with respect to the target mRNA. Serving as the template for sequence-specific gene silencing by the RNAi machinery, the antisense strand of siRNA is also called the guide strand, while the sense strand is known as the passenger strand. 

Chowdhury et al. have recently described an approach to design siRNAs for silencing nucleocapsid phosphoprotein and surface glycoprotein genes of SARS-CoV-2, demonstrating the potential power of siRNA to combat emergent pandemics [11]. Other more advanced application of siRNA has also been described by Zhang et al., indicating the broad application potential of the siRNA technology [12]. An excellent example of achieving imaging-guided and tumor-targeted siRNA delivery and cancer treatment has recently been published by Guo et al. [13], highlighting the promising potential of siRNA for translational biomedicine. Those who are interested in broader applications of siRNA technology are referred to a recent comprehensive review by Weng [14].

There are many potential siRNA molecules that may be able to knock down a specific target mRNA: if there are N nucleotides in the target mRNA, N-20 siRNAs of 21 nucleotides can be designed by sliding a 21 nucleotides sequence along the entire length of the mRNA. However, only a fraction of these candidate siRNAs is highly effective in silencing the target mRNA [15,16]. Applying more than five different siRNAs may lead to the saturation of the RNA-induced silencing complex with the degradation of untargeted genes [17]. Therefore, selecting the few most effective siRNAs from the large number of candidate siRNAs is crucial for maximizing specific gene silencing and minimizing off-target effects. Toward this end, numerous algorithms have been developed to facilitate the rational design of siRNA. 

Two categories of approaches have been published: rule-based methods and machine learning-based methods. The rule-based methods exploited a variety of features that are more easily interpretable, such as the thermodynamic features describing binding free energies and the sequence stability [18,19,20], compositional features describing the occurrences of certain nucleotides at certain positions of the siRNA sequences [16,21,22], secondary structure characteristics of the mRNA target and the siRNA [23,24,25], and uniqueness of the target site [26,27]. These siRNA features have been reviewed [28,29,30], and methods based on these features are intuitive and beneficial to uncovering the fundamental requirements for active siRNA molecules. However, they often produced high levels of false positive predictions when tested on data from external sources [31,32]. More complicated factors are likely involved in RNAi, and cooperative interactions among the various factors may play an important role in determining the efficacy of siRNA-mediated gene silencing. To improve the prediction accuracy and model robustness, more rational approaches involving machine learning methods and multivariate data analysis emerged [33,34,35]. These algorithms have advanced the prediction tools in terms of quantitatively predicting siRNA potency in a genome-wide gene silencing study, and they based their predictions on such features as duplex stability, sequence characteristics, mRNA secondary structure, siRNA secondary structure, and the target site uniqueness. For example, He et al. [36] have described a study of a combination of nucleotide frequency, thermodynamic stability, as well as thermodynamic siRNA–mRNA interaction as a new kind of mixed features. These features work well for natural siRNAs and do not take the chemical structures into account. Jia et al. employed C-features that are based on frequencies of different combinations of nucleotide letters [37]. As with similar features based on nucleotide single-letter representation, frequencies of single-letter combinations do not reflect the chemical nature of nucleotides involved, especially when dealing with chemically modified nucleotides. Ayyagari [38] has recently described an in silico study using existing siRNA predicting services to design potential siRNAs against SARS-CoV2. This article described an excellent example to demonstrate the significance of siRNA prediction in cases of urgent needs. We note that in all these works, no chemical structural features of individual nucleotides (NTs) of a siRNA sequence have been encoded. Thus, they cannot be used to predict the potency of gene silencing mediated by chemically modified siRNA molecules, which provides solutions to many of the challenges facing siRNA therapeutics [39]. Tatabatake et al. demonstrated that chemical modification could improve the nuclease resistance of siRNA molecules, prolonging their activity [40]. Koller et al. suggested that chemical modification could increase the potency of the modified siRNAs [41]. To reduce or avoid immune-mediated and hybridization-dependent off-target effects, careful sequence design is an essential first step, while chemical modifications can provide further protection [39,42,43]. 

Here, we propose a novel approach that describes siRNA molecules in a more physicochemical way, and the models can thus predict the potency of gene silencing triggered by chemically modified siRNAs (as well as by natural siRNAs). While the sequence information of a siRNA is at the core of siRNA-mediated gene silencing, in this approach, we hypothesize that the potency of a siRNA is also strongly dependent on the chemical structure of individual nucleotides that compose a siRNA molecule. Thus, we can describe a siRNA molecule in terms of two crucial characteristics: we first numerically describe each nucleotide (NT) using a set of cheminformatics descriptors, which together should capture its physicochemical properties that might be correlated with molecular interactions, and then the whole siRNA sequence is described by simply concatenating all the descriptors in the order of the siRNA’s sequence. In contrast, current literature methods do not encode the chemical nature of each nucleotide; instead, they simply use the one-letter representation of different nucleotides, e.g., counting the frequencies or thermodynamic stability based on local pairs of nucleotides. Since there are chemical similarities/differences among the nucleotides, encoding the chemical nature of each of the composing nucleotides can offer additional information to reflect the chemical similarity and dissimilarity when comparing different strands of siRNAs. 

The partial least square (PLS) regression [44] method is employed to develop statistical models of gene silencing potency. For regular siRNAs, the Huesken dataset (with 2431 siRNAs) [33] was used to perform model building, validation, and comparative analysis. The results from this study were comparable with or superior to those from the Huesken paper [33] in terms of the Pearson coefficients; to model the chemically modified siRNAs, the Bramsen dataset (with 48 chemically modified siRNA molecules) [45] was used to build and validate the models. The predictive power of the resulting models has reached 0.64–0.65 (i.e., the Pearson coefficients ranged from 0.81 to 0.82). In the following sections, we first detail the methodology for descriptor generation and the modeling workflow, followed by results and discussion.

## 2. Materials and Methods 

### 2.1. The Huesken Dataset

**Figure 1:**
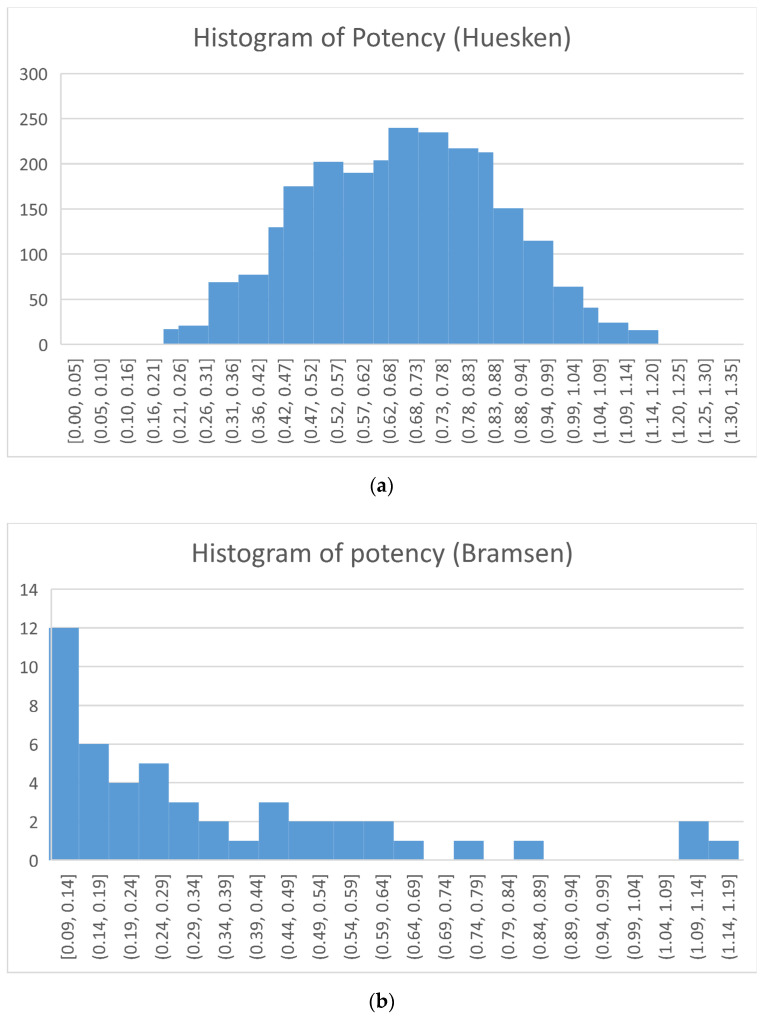
Histograms of gene silencing potencies: (**a**) the Huesken dataset and (**b**) the Bramsen dataset.

To build and validate models for the potency of siRNAs composed of natural nucleotides, 2431 siRNA sequences (antisense format) of the Huesken dataset [33] were used. The dataset is considered to be the first landmark data publicly available for siRNA gene knockdown experiments, targeting 34 different mRNAs. Both 19 NT sequences without the overhangs (2 NT), as well as the 21 NT sequences with overhangs, are available. Considering that the overhangs have an effect on the potency of gene silencing, the 21-NT sequences were used in building models in this study. The gene knockdown potency was expressed as normalized. Figure 1a shows the distribution of the gene silencing potency by the siRNAs in this dataset, indicating a wide potency range. 

Eight subsets of sequences were created according to Huesken et al. [33] for comparison purposes. These subsets are named All (2182), All human (1744), Human E2s (1229), Rodent (438), Random all (1091), Random all (727), Random all (545), and Random all (218). These subsets were used as training sets to build corresponding models. Four subsets were also created for use as the test sets, and they are All (249), All human (198), hE2 (139), and Rodent (51). Note that the numbers in the parentheses refer to the number of siRNA sequences in that subset.

### 2.2. The Bramsen Dataset

In order to predict the potency of siRNAs consisting of chemically modified nucleotides, 48 siRNA sequences targeting eGFP from Bramsen et al. [45] were used to build and evaluate models. There are 21 different types of chemical modifications involved in this dataset. The modifications can be broadly categorized into 2′-substituted RNAs, 4′-modified RNAs, locked RNAs, and RNAs with radical modifications of the ribose sugar ring [45]. For each modified siRNA molecule, the 21 NT antisense sequences with overhangs were used in this study. The gene knockdown potency was expressed as eGFP level, where a larger eGFP level indicates a poorer potency. Figure 1b shows the distribution of the gene silencing potency by this set of chemically modified siRNAs; clearly, the potency of this dataset is less diverse compared with the Huesken data. 

### 2.3. Overall Workflow for the Predictive Modeling of siRNA Potency

**Figure 2:**
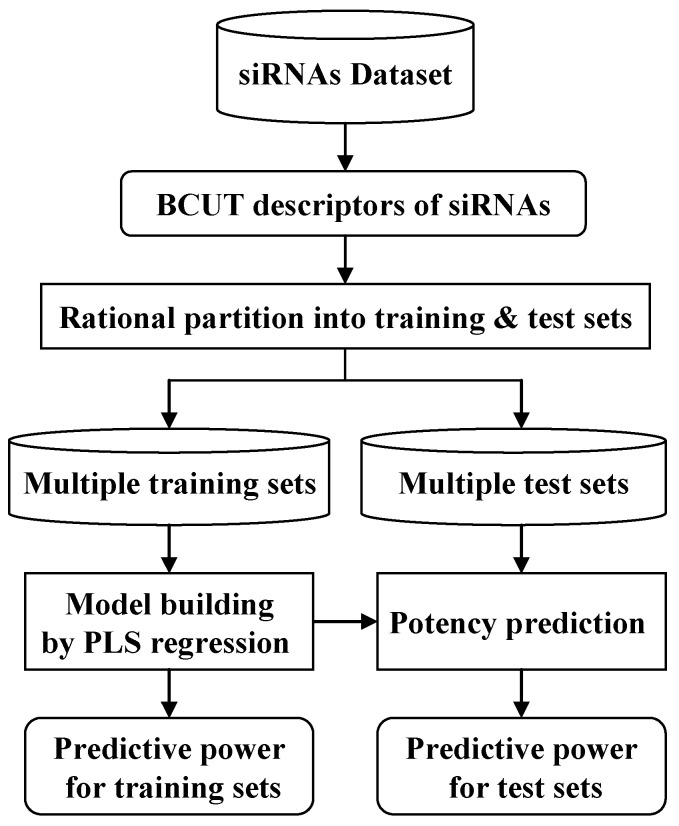
The workflow for model building and model validation. Rational design of training and test sets were achieved using ART-2a to cluster the dataset. Partial least square (PLS) models were built upon each of the training sets. Models were then used to predict test set siRNAs, and the Pearson *r* (and *r*^2^ for the chemically modified siRNA dataset) were obtained for all the test sets.

The overall workflow for building predictive models for siRNA potency is shown in Figure 2. It involves the following major steps: (1) preparation of the siRNA dataset, where all the structures of the individual nucleotides involved are built and processed utilizing the MOE program, while the siRNA antisense sequences and the corresponding potency values are verified; (2) generation of the BCUT descriptors for each of the siRNA molecules in the dataset; (3) rational partitioning of the dataset into pairs of training and test sets; (4) building predictive models utilizing the partial least square (PLS) procedure as implemented in MOE; (5) validating the models, where each model is tested using the corresponding test set. 

It is worth noting that in some implementations of PLS, one uses LOO (leave-one-out) as the way to select the optimum number of principal components (PC) for developing final models. Alternatively, as implemented here in MOE, it is customary to scan the number of principal components (PC) to find the optimum models and use a second set as the validation set. In recent years, a second “external” set is often employed to further validate the models; however, it has been an accepted practice in the QSAR community to employ one training vs. one test set [46], which is employed in this study. 

Details of siRNA descriptor generation, the rational design of the pairs of training and test sets, model building, and validation procedures are as follows. 

### 2.4. Generation of the BCUT Descriptors for Each siRNA Molecule

Specifically, we choose the 12 BCUT descriptors [47,48] implemented in the MOE software (Chemical Computing Group, Montreal, CA, USA), describing the charge distribution, hydrophobic property distribution, and polar property to characterize the chemical structure of each individual nucleotide. A 21-NT siRNA molecule is then numerically described by 252 BCUT descriptors. 

One straightforward approach to describing a siRNA molecule is to calculate typical cheminformatics descriptors used to describe small organic molecules. However, it often fails to capture the sequential information of polymeric molecules such as peptides and nucleotides-the repeated occurrence of the same chemical building blocks composing a polymeric molecule often leads to the degeneracy of descriptors. This issue had been discussed by Sandberg [49] as well as by Jonsson [50,51]. Thus, our approach to characterizing siRNA molecules is similar to those by Sandberg and Jonsson in their respective description of peptides and DNA molecules. The basic concept is that a polymeric molecule can be numerically characterized by molecular descriptors of its composing building blocks. Thus, a siRNA molecule can be described by molecular descriptors of its composing nucleotides (A, U, G, C, T, and their different chemical modifications). Specifically, each nucleotide position in a siRNA sequence can be translated into the corresponding descriptor values for the chemical structure of the NT. 

**Figure 3:**
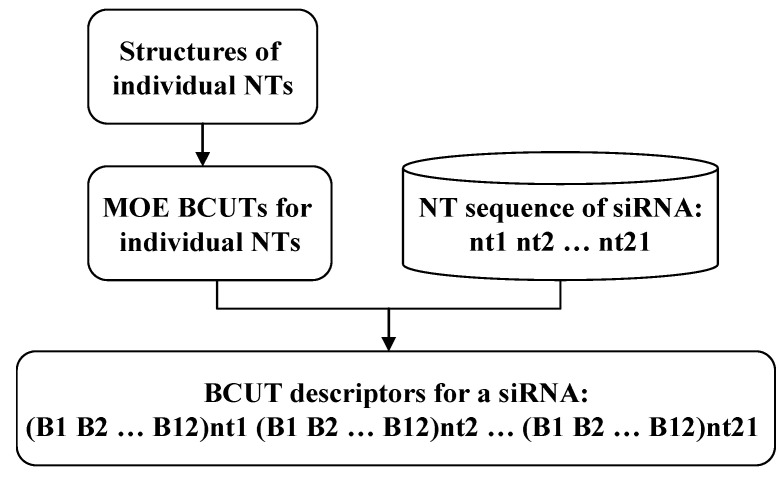
Descriptor generation for a siRNA molecule.

First, we calculate the 12 BCUT molecular descriptors for each of the nucleotides involved: for the Huesken dataset, BCUT for five natural nucleotides was calculated, while for the Bramsen dataset, BCUT for 98 chemically modified nucleotides was calculated employing the MOE program. The original siRNA sequences for both datasets and the corresponding BCUT values for involved nucleotides are provided in the Supplementary Materials. Then by concatenating the BCUT descriptors for composing nucleotides on the basis of a given siRNA sequence, the descriptors for a siRNA molecule are obtained. The procedure is depicted in Figure 3. 

**Rational Design of Pairs of Training and Test Sets**. The basic idea behind the rational design of a pair of training and test sets is that molecules in the test set should be properly represented by the molecules in the training set. Golbraikh et al. used what they called the sphere exclusion algorithm to select molecules for training and test sets [52,53]. Here, we adopted a well-validated clustering algorithm called ART-2a developed by Carpenter [54]. One advantage of the ART-2a algorithm is that it keeps updating the centroid of each cluster so that the centroid of each cluster faithfully represents the molecules in that cluster. In this work, the whole set of siRNAs is first converted to their numerical representation as described above. The data matrix was subject to ART-2a clustering. The vigilance parameter was adjusted so that the desired number of multimember clusters was obtained: 278 multimember clusters and 9 multimember clusters were obtained for the Huesken dataset and the Bramsen dataset, respectively. The test set molecules were then randomly picked to cover all the above clusters on the condition that the most potent siRNA and the least potent siRNA were not included in the test set. After the test set siRNAs were selected, the remaining siRNAs were used as the corresponding training set. The main reason for using chemical structure-based clustering for training–testing set design is to ensure the structural diversity of the siRNAs in the training set. Chemical structure-based diversity design is critical for training and testing set design, as discussed by Golbraikh [53].

To demonstrate the statistical confidence of the models, this process was repeated several times; as a result, multiple pairs of training and test sets were generated for model building and model validation: 30 pairs for the Huesken dataset and 30 pairs for the Bramsen dataset. 

**Model Building Technique**. Many different machine learning algorithms have been used in the modeling of gene silencing potency. The most popular ones are linear regression [35], Support Vector Machine (SVM) [26], Artificial Neural Network (ANN) [33], and decision tree [20]. In this study, the partial least square (PLS) regression method was selected to build the predictive models to avoid any potential overfitting issues facing many of the above algorithms. It generalizes and combines features from principal component analysis (PCA) and multiple linear regression analysis. This prediction is achieved by extracting from the predictors a set of orthogonal factors (a.k.a. latent variables or principal components) which have the best predictive power. The advantages of PLS include the ability to handle multi-collinearity among the predictors, robustness in noisy and missing data, and reduction in overfitting when the number of predictors gets too large. In our studies, the number of principal components was set as an adjustable variable scanned in each model development to avoid both overfitting and under-fitting issues for any given dataset. 

**Model Validation Strategies**. Validation is a crucial aspect of quantitative predictive modeling. We employed five analyses to ensure the quality of the built models as follows:

1. ***Correlation strength and predictive power***. The correlation coefficient *r* (i.e., Pearson r) was computed to measure the correlation strength, and the predictive *r*^2^ was used to measure the prediction power. The values of model development *r* (or *r*^2^) were calculated on the basis of the actual potency and model-predicted potency for the training set siRNAs. They served as the necessary requirement for a reliable quantitative model. Testing *r* (*r*^2^) was calculated on the basis the actual potency and model-predicted potency for the test set molecules. The value of *r* (or *r*^2^) was viewed as another necessary requirement for a suitable predictive model [46]. Equation (1) was used to determine the value of *r*, and Equation (2) was used to compute the value of *r*^2^, applicable to both training and test sets. In the equations, *y_i_* and *p_i_* are the actual and predicted potencies, respectively; $\bar{y}$ and $\bar{p}$ are the means of the *y_i_* and *p_i_*, respectively. N is the number of siRNA molecules.

(1) $$r=\frac{\overset{N}{\underset{i=1}{\sum }}\left(y_{i}-\bar{y}\right)\left(p_{i}-\bar{p}\right)}{\sqrt{\overset{N}{\underset{i=1}{\sum }}{\left(y_{i}-\bar{y}\right)}^{2}\overset{N}{\underset{i=1}{\sum }}{\left(p_{i}-\bar{p}\right)}^{2}}}$$

(2) $$r^{2}=1-\frac{\overset{N}{\underset{i=1}{\sum }}{\left(y_{i}-p_{i}\right)}^{2}}{\overset{N}{\underset{i=1}{\sum }}{\left(y_{i}-\bar{y}\right)}^{2}}$$

2. ***The effect of the number of principal components on the predictive models***. The principal component analysis technique was used to extract a set of orthogonal factors that afford the best predictive power. The proper number of principal components is dependent on the size of the training set and the relationship between the descriptors. To establish the best models for a given dataset, we scanned the number of principal components to find the optimal numbers for use in the model.
3. ***The effect of data partitioning on the predictive models***. To ensure the predictive power of the built models, we rationally split/partition the dataset into training and test sets. The training set was used to establish the models, and the corresponding test set was used to validate the models. The molecules in the test set were not involved in the model building; thus, the predictive *r*^2^ calculated on the basis of the test set will more objectively indicate the true predictive ability. Different partitioning of training and test sets could give rise to models with different predictive powers, especially when the dataset is small, and we performed a series of computational experiments to find the best models.
4. ***The effect of training set size on the predictive models***. The predictive power is strongly dependent on the size of the training set. Thus, different percentages of the original dataset were selected to be used in the training set. The ideal case was to use the least number of siRNAs to develop models, which are then used to predict the greatest number of siRNAs. We used the Huesken dataset to demonstrate this where 1%, 2%, 3%, …, and 90% of the whole dataset were used as the training set; and the models were used to predict the potency of 99%, 98%, 97%, …, and 10% of the remaining siRNAs, respectively.
5. ***Effect of random shuffling on model development.*** The predictive models built should faithfully reflect the intrinsic relationship between the descriptors and the gene silencing potency for a given dataset. A random dataset should not result in a predictive model. To prove this, we first randomly shuffled the potencies among the whole dataset, and then the models were built from these scrambled datasets. A different number of principal components was used to perform the PLS (partial least square) modeling. We should expect a dramatic decrease in the predictive power of the models built on the scrambled dataset.

In addition, when models are used to predict truly unknown molecules, the applicability domain should be applied before the prediction is made. For example, one should employ the applicability domain as one of the filters, as recommended in the standard workflow protocol advocated by Tropsha [55].

## 3. Results and Discussion

### 3.1. Modeling of the Huesken Dataset

***BCUT Descriptors for Natural Nucleotides.*** The chemical structures of the five natural nucleotides (A, C, G, U, and T) were sketched; and the 12 BCUT descriptors were calculated: BCUT_PEOE_0, BCUT_PEOE_1, BCUT_PEOE_2, BCUT_PEOE3, BCUT_SLOGP_0, BCUT_SLOGP_1, BCUT_SLOGP_2, BCUT_SLOGP_3, BCUT_SMR_0, BCUT_SMR_1, BCUT_SMR_2, and BCUT_SMR_3. The BCUT_PEOE descriptors describe the charge distribution of a molecule. They are calculated from the eigenvalues of a modified adjacency matrix; the BCUT_SLOGP and BCUT_SMR descriptors characterize the hydrophobic property and the polarizability of a molecule, respectively. These two sets of descriptors are determined from the eigenvalues of their respective modified adjacency matrixes. Table 1 shows the values of the BCUT descriptors for the five natural nucleotides. Because the 12 BCUT descriptors represent the main features involved in intermolecular interactions, we replace the nucleotides in the siRNA sequences with their 12 BCUT descriptors; the numerical descriptors coupled with the multivariate analytical tool (PLS) should capture any correlation that might exist in the dataset.

**Figure 4:**
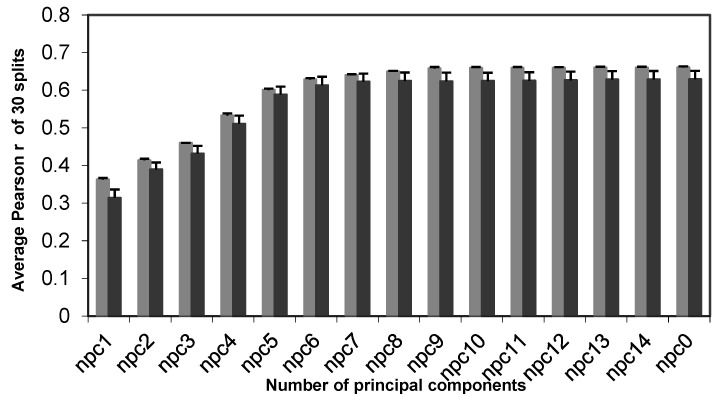
The effect of splits on models for the Huesken dataset. The mean and the standard deviation of the Pearson correlation coefficients are calculated among 30 rational splits. The number of siRNAs in the training set is 2153, and the number of siRNAs in the test set is 278. Pearson *r* for both the training set (in gray) and test set (in dark gray) are shown.

***The Effect of Dataset Partitioning/Splitting on the Models.*** To avoid the bias of individual rational splitting of the dataset into training and test set, we performed 30 rounds of rational splitting; 30 pairs of training and test sets were thus generated. For each training set, we developed 15 PLS regression models corresponding to the number of principal components being “all”, 1, 2, 3, 4, 5, 6, 7, 8, 9, 10, 11, 12, 13, and 14, respectively. Each of the 15 models was then used to predict the potencies of siRNAs in the corresponding test set. For comparison with the original Huesken results, we used the correlation coefficient *r* as the measure for the quality of models. For each number of principal components, the mean and the standard deviation of the Pearson correlation coefficients were calculated from 30 rational splits. As shown in Figure 4, the standard deviations were very small for each case, meaning that each of the 30 splits gives consistent results. Table 2 lists the related statistics to detail the information in Figure 4. The best model reported by Huesken et al. had a Pearson coefficient of 0.66 for one specific pair of training and test sets. This falls within our resulting range of 0.58–0.68 (for the test set), indicating that our method produced comparable models to Huesken’s model BioPredsi [33]. According to a recent benchmark study by Matveeva et al. [35], BioPredsi was the best model tested thus far. Therefore, our new approach has demonstrated a performance similar to the best model reported in the literature.

In all the reported cases, the Pearson correlation coefficient *r* has been used as the quality indicator. It has been reported [33] that the potency data experimentally tested on two different plates of siRNAs had a Pearson correlation coefficient of about 0.70. Thus, all the models with Pearson correlation coefficients of >0.60 were considered reasonable models in the context of these experiments.

**Figure 5:**
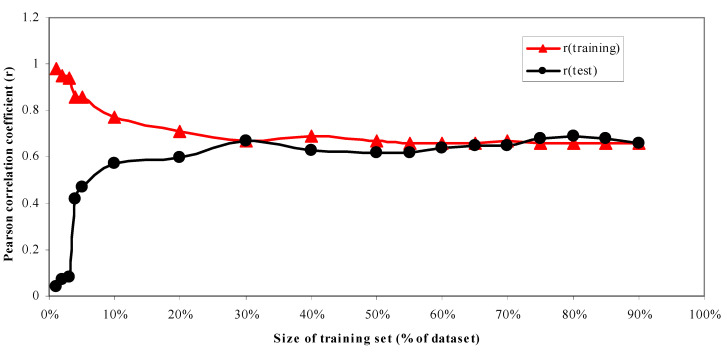
Pearson correlation coefficients as the function of the size of the training set. Both training set *r* (in red) and the corresponding test set *r* (in black) are shown.

***The Effect of Training Set Size on Model Quality.*** For a dataset as large and diverse as the Huesken dataset, there should be greater flexibility for the size of the training set (as well as the size of the corresponding test set) to be used. We set the training set size as the percentage of the whole dataset to be 1%, 2%, 3%, 4%, 5%, 10%, 20%, 30%, 40%, 50%, 55%, 60%, 65%, 70%, 75%, 80%, 85%, and 90%, respectively, to develop the models and predict the corresponding 99%, 98%, 97%, 96%, 95%, 90%, 80%, 70%, 60%, 50%, 45%, 40%, 35%, 30%, 25%, 20%, and 15% of the whole dataset. We set the number of principal components to be “all” for all the models for this experiment. The Pearson correlation coefficients of the models were calculated both for the training and the test sets. Figure 5 shows that if 20% or more of the dataset was taken as the training set to build the models and predict the remaining 80%, the resulting Pearson correlation coefficients were all greater than 0.60. All the models were regarded as acceptable.

**Figure 6:**
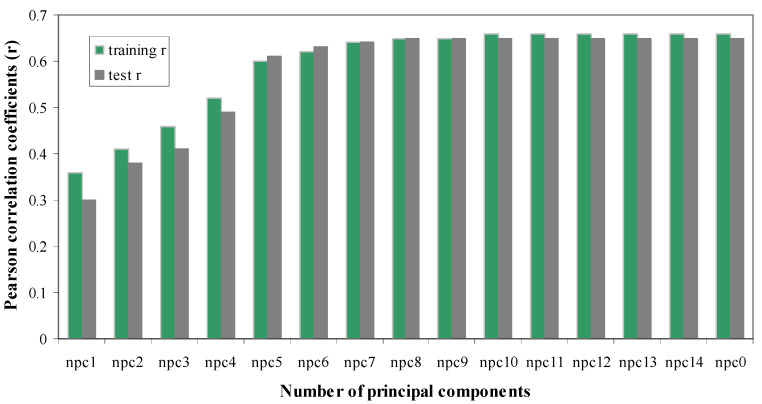
Pearson *r* as the function of the number of principal components. There are 2153 and 278 siRNAs in this training set and test set, respectively. Pearson *r* was shown for both the training set (in green) and the test set (in gray).

***The Effect of Number of Principal Components on Model Quality.*** To obtain the best models for the Huesken dataset, we set the numbers of principal components of PLS models to be “all”, 1, 2, 3, 4, 5, 6, 7, 8, 9, 10, 11, 12, 13, and 14 to build models. These models were used to predict the potency of siRNAs in the corresponding test sets. To be consistent with the original work, we used 2153 siRNAs as the training set, and the remaining 278 siRNAs were used as the test set. Pearson *r* was computed for both the training set and the test set to measure the quality of the models. One pair of training and test sets was shown in Figure 6, while the other 29 pairs of training and test sets gave similar results. If the number of principal components is “all” or greater than 10, all the models were of acceptable quality. If the number of principal components was “all” or greater than 5, all the models also had Pearson *r* greater than 0.60. 

**Figure 7:**
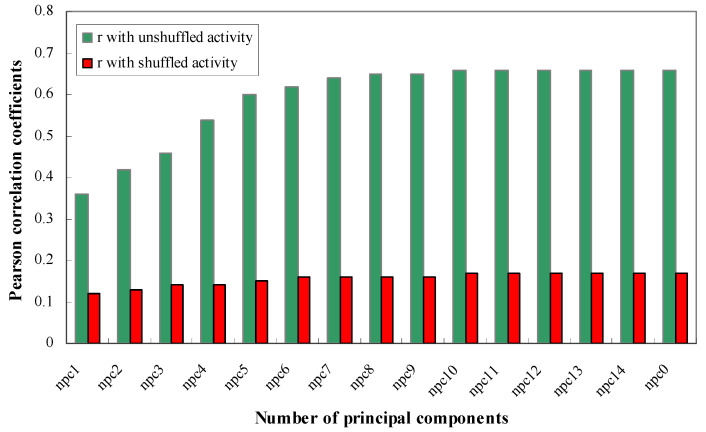
Effect of randomly shuffled siRNA potencies on the models. The Pearson *r* values are shown for models built on both the original Huesken dataset (in green) and the scrambled dataset (in red).

***The Effect of Random Shuffling on Modeling.*** To ensure that the models faithfully reflect the intrinsic relationships between the descriptors of siRNA and the gene silencing potency, rather than finding spurious correlations, we randomly shuffled the potencies of the Huesken dataset. We tried to build models based on the scrambled dataset. Fifteen models were built with the number of principal components fixed to “all”, 1, 2, 3, 4, 5, 6, 7, 8, 9, 10, 11, 12, 13, and 14, respectively. As shown in Figure 7, the scrambled dataset did not result in any reliable models—the values of Pearson *r* for the scrambled data had significantly decreased (by 66–75%) compared with the original dataset. These results further supported the conclusion that our models were not spurious ones.

**Figure 8:**
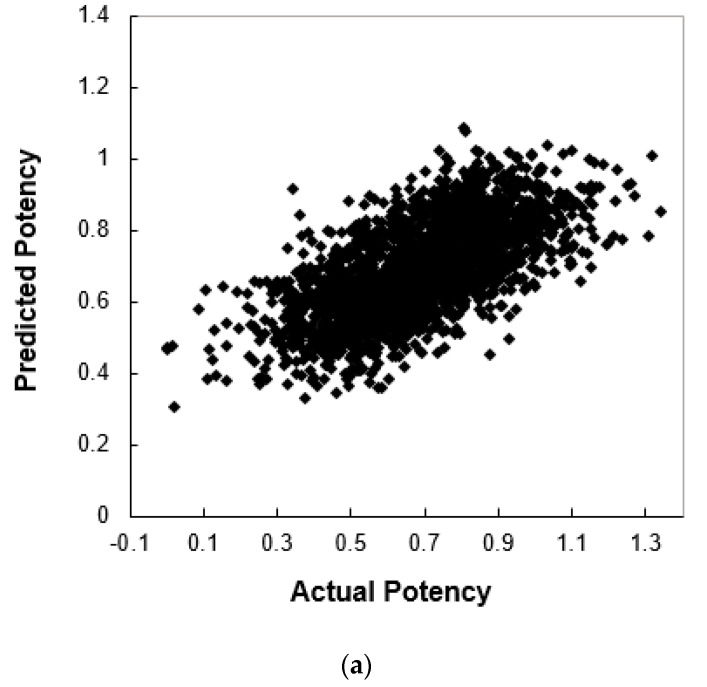

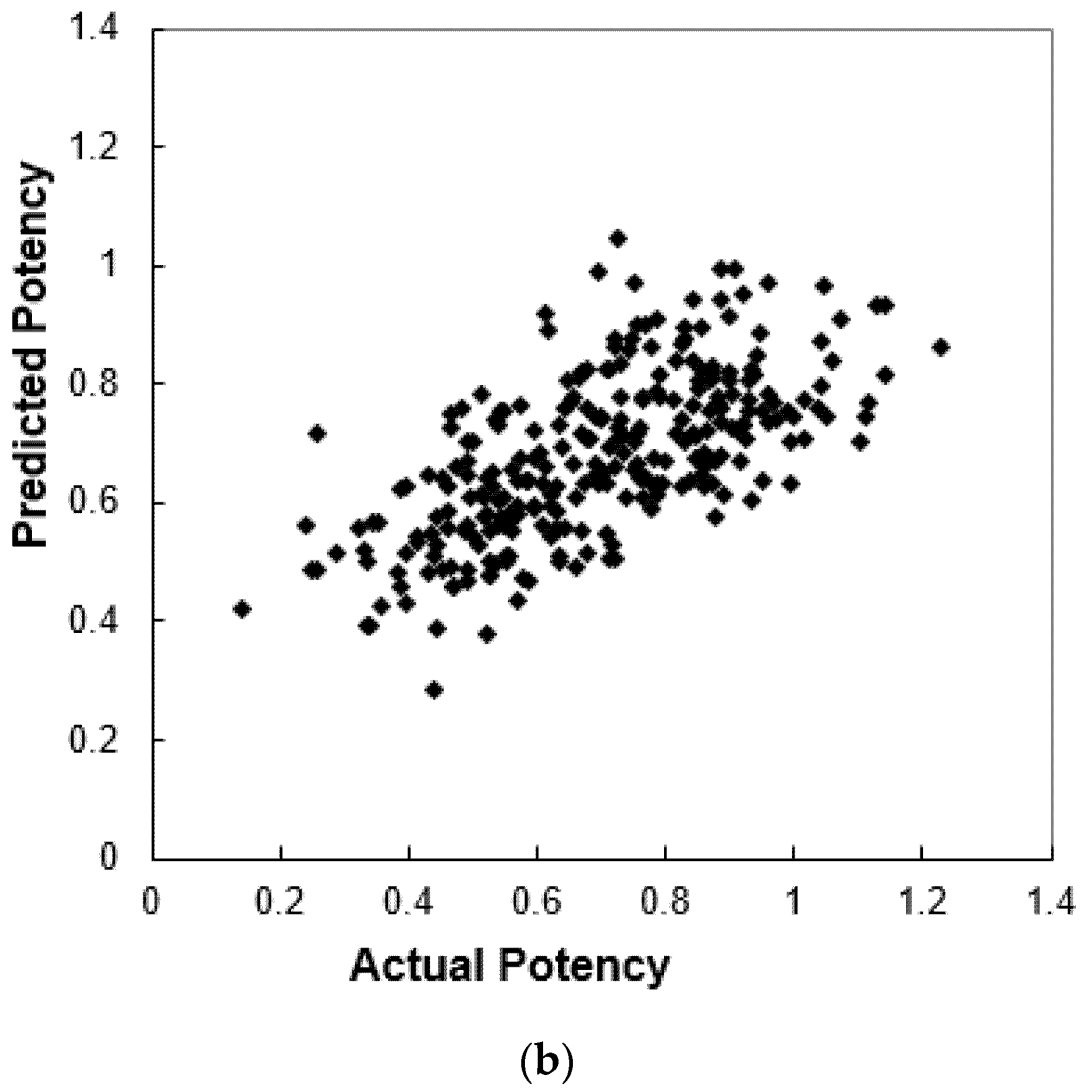
Model-predicted potency versus the actual potency for the Huesken dataset: (**a**) the training set and (**b**) the test set.

***Predictive Models Have Been Obtained.*** To demonstrate the quality of the resulting models, the scatter plots of actual against predicted potencies are shown in Figure 8a (of the training set) and Figure 8b (of the test set) for one of our final models. As mentioned in the discussion on data partitioning effect on model quality, other models also gave similar results with a standard deviation of 0.003 for the training sets and 0.02 for the test sets. The Pearson *r* is 0.67 for both the training set and test set, as shown in Figure 8. The RMSE (root mean square error) [56] values for both the training and test sets are 0.15.

To compare the performance of our new method with that of the Huesken [33], we performed modeling of the exact same set of subsets as Huesken. Specifically, the same subsets of training and test molecules reported by Huesken were used to conduct our studies. The subset specification is given in Materials and Methods. Some of the subsets were based on species, such as human vs. rodent; others were based on genes (E2 sequences) as well as random selections of siRNAs. The results are shown in Table 3. The Pearson correlation coefficients in parentheses are quoted from Huesken or comparison. In some cases, the Huesken model (i.e., BioPredsi) performed slightly better; in other cases, our method outperformed BioPredsi. Overall, our approach is comparable with or superior to BioPredsi [35]. 

The predictive models developed on the basis of the Huesken dataset can be used in the virtual screening of potential siRNA molecules of natural nucleotides for a given target mRNA. Those siRNA molecules that have passed the Watson–Crick pairing with the target mRNA and are scored well by our models could be prioritized as top choices for focused RNAi experiments. These models should be useful in designing potential siRNA molecules or therapeutics against specific genes under study. 

***Sequence-based Features and Critical BCUT Descriptors.*** As mentioned in the introduction, we hypothesized that both the sequence and the chemical structures of the composing nucleotides play a significant role in siRNA gene silencing potency. Thus, in addition to accurately predicting the potencies of siRNAs, we also proposed an analysis that can provide more insights into siRNA design. To be more specific, we performed relative importance analyses regarding the 21 nucleotide positions and the 12 different BCUT descriptors, respectively. We calculated the relative importance for the *i*-th descriptor as the normalized absolute value of the regression coefficients for the *i*-th descriptor in the PLS models. We calculated the relative importance for one of the 12 BCUT descriptors as an averaged value among 30 rational splits and the 21 nucleotide positions. 

**Figure 9:**
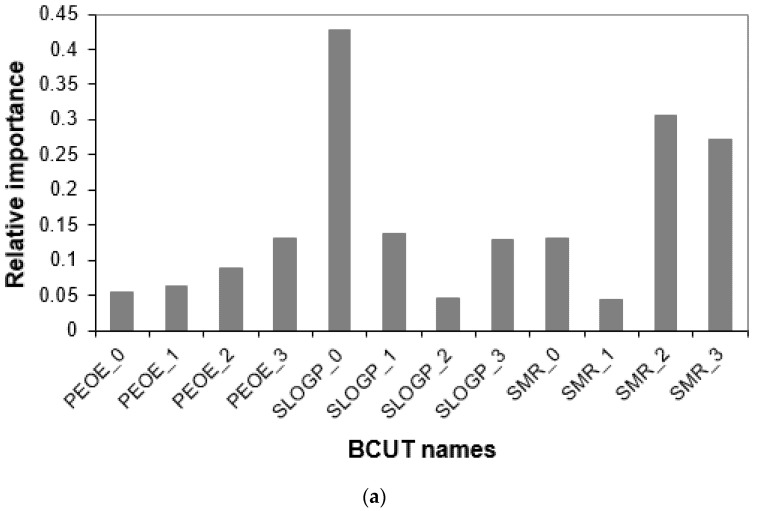

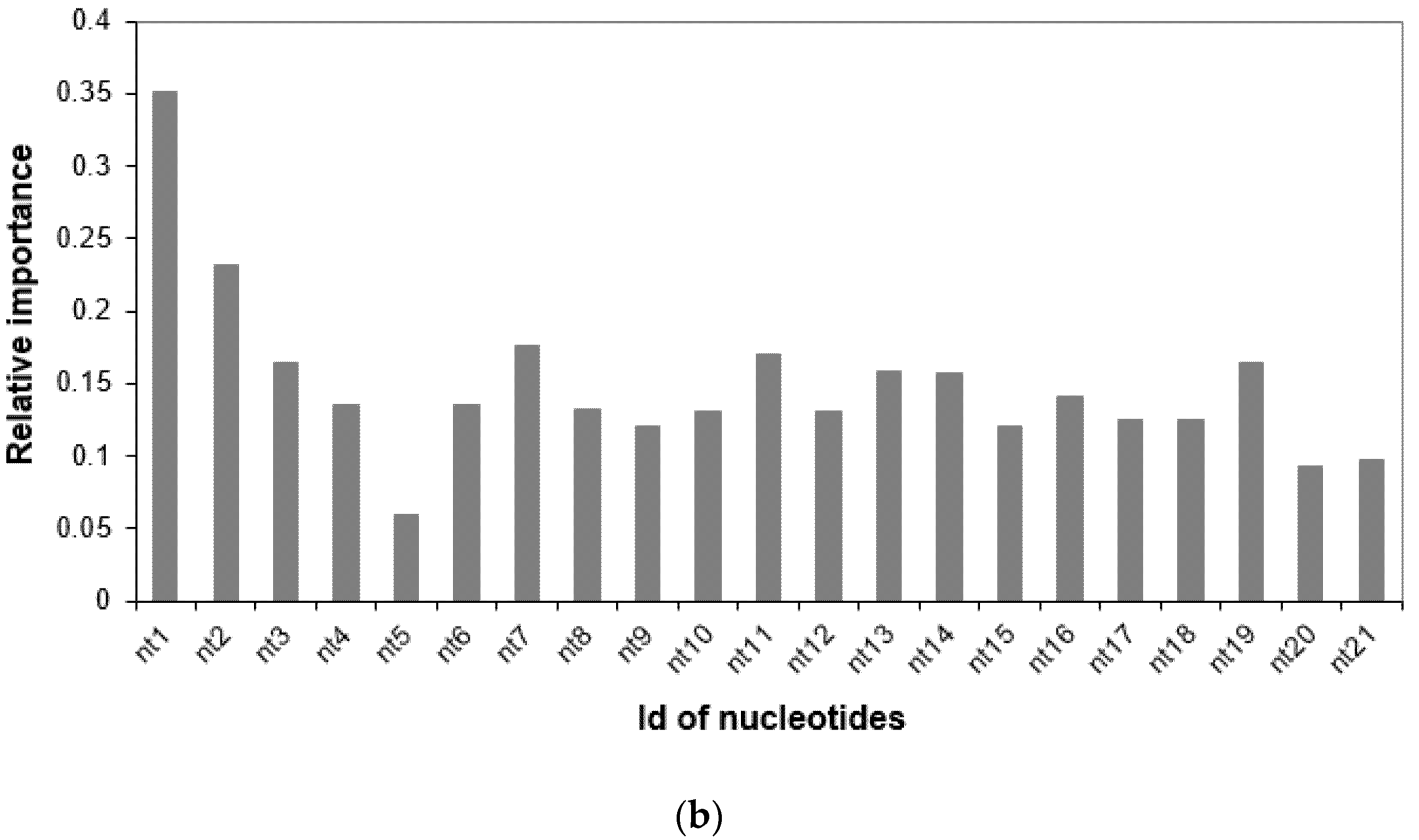
The relative importance of BCUT descriptors and nucleotide positions: (**a**) the BCUT descriptors and (**b**) the nucleotide positions.

Figure 9a shows the relative importance of the 12 BCUT descriptors for siRNAs of natural nucleotides. The charge distribution may be less important compared with hydrophobic distribution and polar property distribution of the natural nucleotides. Likewise, the relative importance for one of the nucleotide positions is averaged among 30 rational splits and 12 BCUT descriptors of the nucleotide. Here, nt1, nt2, …, and nt21 represent the nucleotides in the first, second, …, and 21st positions of a siRNA, respectively. The nt20 and nt21 are the overhangs. Figure 9b depicts the relative importance among the 21 composing nucleotides. It is consistent with previous publications [36,57] that the overhang has a noticeable contribution to the potency even though their contributions are not among the highest (see Figure 9a). Specifically, the descending order of relative importance among the 21 nucleotide positions is: nt1, nt2, nt7, nt11, nt19, nt3, nt14, nt6, nt4, nt12, nt17, nt18, nt15, nt9, nt21, nt20, and nt5. 

### 3.2. Modeling of the Bramsen Dataset

Following the same approach detailed in Materials and Methods, we performed data preparation, model development, and model validation for the Bramsen dataset. To our best knowledge, this is one of the first modeling work for gene silencing potency by siRNAs of chemically modified nucleotides. Since this dataset is relatively small (48 chemically modified siRNAs), we decided to use more stringent criteria to measure the quality of models. Instead of the Pearson correlation coefficient alone, we also used predictive *r*^2^ to judge the quality of the models. Specifically, we selected the models based on criteria of both Pearson *r* and predictive *r*^2^ equal to or greater than 0.60 for the test sets.

**Figure 10:**
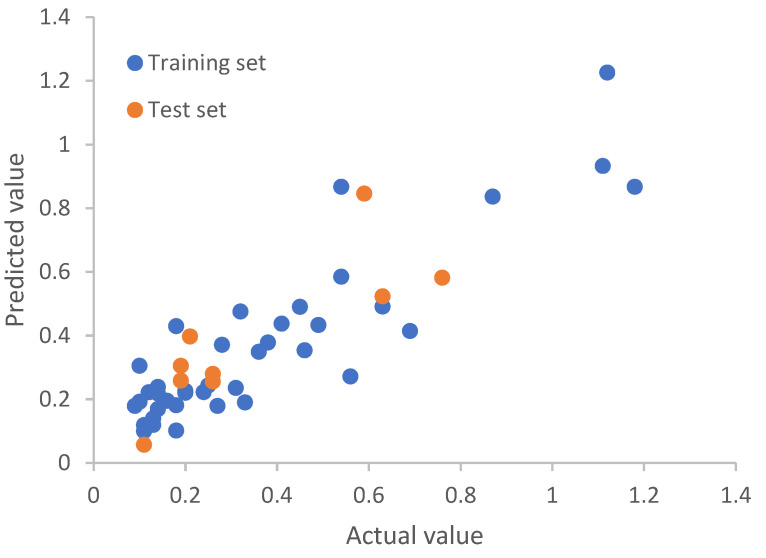
Model-predicted potency value versus the actual value by top model 1 for the Bramsen dataset.

**Figure 11:**
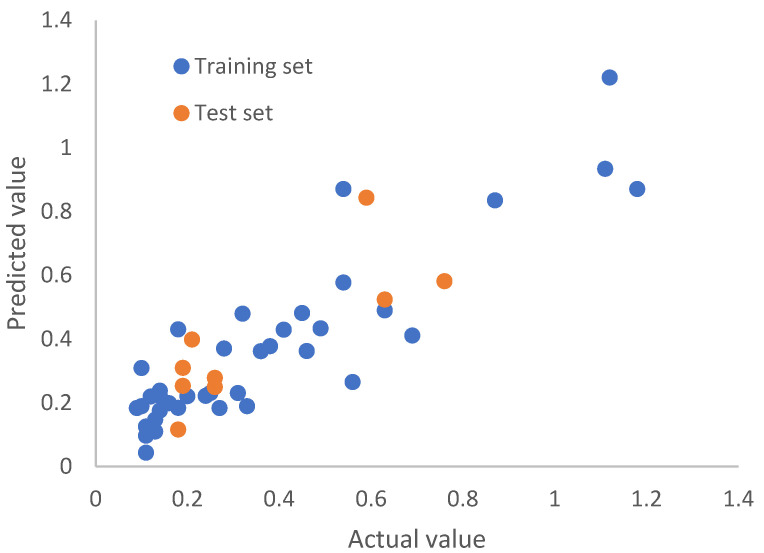
Model-predicted potency value versus the actual value by top model 2 for the Bramsen dataset.

**Figure 12:**
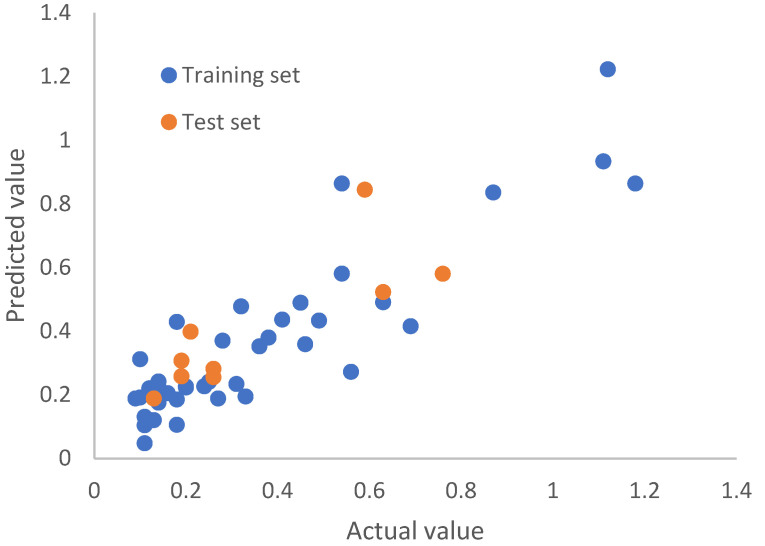
Model-predicted potency value versus the actual value by top model 3 for the Bramsen dataset.

Following the same protocol as that for the Huesken dataset, we replaced each nucleotide with its 12 BCUT descriptors, resulting in 252 descriptors for a siRNA molecule. By rational splitting, we generated 30 pairs of training and test sets. The number of siRNAs in each training set and test set was 39 and 9, respectively. Given that there are 252 descriptors and 39 training set molecules, the number of principal components of PLS modeling will have a strong effect on the quality of models. To find models of best predictive power, for each of the 30 training sets, we examined the number of principal components (from 1, 2, 3, 4, 5, 6, 7, 8, 9, and 10) to build the models, and the corresponding test set was used to validate the models. For the Bramsen dataset, if the number of principal components is smaller than 4, the resulting models were not predictive by our criteria, although the Pearson *r* and the predictive *r*^2^ were good for the training set. When the number of principal components employed was too large, there was overfitting. After careful examination, we selected three reliable predictive models, each of which has a number of principal components of 4. The Pearson *r* for the test set was 0.82, 0.82, and 0.81 for model 1, model 2, and model 3, respectively. The RMSE values for the test set were 0.135, 0.136, and 0.136, respectively. The predictive *r*^2^ for the test set was 0.63, 0.62, and 0.60 for model 1, model 2, and model 3, respectively. Figure 10, Figure 11 and Figure 12 are the scatterplots for model 1, model 2, and model 3, respectively. Collectively, Figure 10 through Figure 12 show that the three models for chemically modified siRNA have both good training and testing quality. The original and predicted values for the three splits, as well as the source code for conducting rational splitting, are provided in the Supplementary Materials. On the basis of these results, we believe that our new approach could play a fundamentally important role in modeling gene silencing of chemically modified siRNA molecules, and it could facilitate the development of therapeutic siRNAs based on modeling chemically modified siRNA molecules. 

## 4. Conclusions 

To summarize, we have developed a new approach for quantitatively predicting gene silencing potency by siRNAs. By characterizing a siRNA molecule using both sequence information and the chemical structures of the composing nucleotides, the new approach has overcome the drawbacks of existing methods that cannot model the potency of gene silencing triggered by chemically modified siRNAs. Our approach has laid a general foundation for quantitative modeling of siRNA gene silencing potency, both natural and chemically modified. This new numerical description of siRNA molecules coupled with PLS (partial least square) affords predictive models of a dataset of 48 chemically modified siRNAs judged by both the Pearson correlation coefficient *r* and predictive *r*^2^ obtained for both training and test sets. To demonstrate the general applicability of the new approach to siRNA modeling, we also built predictive models for a larger dataset with 2431 natural siRNA molecules. The performance of our models was comparable with or superior to that of one of the best models reported in the literature. The robustness of this modeling approach to siRNA-mediated gene silencing has also been established by a series of validation strategies that highlighted the effects of the number of principal components, rational splitting, and the training set size on the quality of models. To our best knowledge, a Web-based prediction tool [58] has been published that used nucleotide compositional patterns (but not chemical structures of nucleotide) as descriptors for the chemically modified siRNA modeling. Our new method is the first attempt to introduce *cheminformatics* descriptors to the modeling of chemically modified siRNA potency. We emphasize that our approach is complementary to other methods in that cheminformatics descriptors can capture the nature of nucleotide chemical structures as opposed to other methods describing nucleotides as arbitrary alphabets—the BCUT cheminformatics descriptors could capture the similarities and differences between different nucleotides in terms of their chemical and physical properties. Because chemical modification provides solutions to many of the challenges facing siRNA design, our new approach can be used to facilitate chemically modified siRNA design for therapeutic purposes.

## Figures and Tables

**Table 1:** MOE BCUT descriptors of natural nucleotides.

NT	PEOE_0	PEOE_1	PEOE_2	PEOE_3	SLOGP_0	SLOGP_1	SLOGP_2	SLOGP_3	SMR_0	SMR_1	SMR_2	SMR_3
A	−2.08	−0.62	0.72	2.13	−2.32	−0.60	0.76	2.19	−1.75	−0.40	0.96	2.45
C	−2.26	−0.49	0.58	2.21	−2.52	−0.44	0.63	2.10	−1.91	−0.38	0.68	2.54
G	−2.26	−0.66	0.73	2.30	−2.63	−0.60	0.77	2.23	−1.92	−0.49	0.92	2.60
U	−2.30	−0.56	0.55	2.29	−2.61	−0.51	0.66	2.10	−1.97	−0.38	0.69	2.60
T	−2.36	−0.49	0.49	2.37	−2.62	−0.34	0.60	2.34	−2.06	−0.31	0.59	2.66

**Table 2:** Model statistics (Pearson *r*) for 30 pairs of training and test sets of the Huesken dataset.

Dataset	Mean	Standard Deviation	Maximum	Minimum
Training	0.66	0.003	0.67	0.65
Test	0.63	0.02	0.68	0.58

**Table 3:** Pearson correlation coefficients for different training and test sets of the Huesken set.

Training Sets (21-NT) ^b^	Test Sets (21-NT)			
	All (249)	All-human (198)	hE2 (139)	Rodent (51)
All (2182)	0.65 (0.66) ^a^	0.62 (0.63) ^a^	0.62 (0.63) ^a^	0.76 (0.77) ^a^
All-human (1744)	0.64 (0.65)	0.60 (0.61)	0.60 (0.62)	0.75 (0.76
Humans-E2s (1229)	0.64 (0.65)	0.6 (0.62)	0.6 (0.62)	0.75 (0.76)
Rodent (438)	0.61 (0.55)	0.6 (0.54)	0.59 (0.53)	0.63 (0.57)
Random-all (1091)	0.64 (0.65)	0.60 (0.62)	0.60 (0.61)	0.76 (0.75)
Random-all (727)	0.64 (0.65)	0.62 (0.63)	0.63 (0.63)	0.72 (0.76)
Random-all (545)	0.60 (0.62)	0.59 (0.60)	0.59 (0.60)	0.66 (0.70)
Random-all (218)	0.52 (0.47)	0.48 (0.47)	0.46 (0.46)	0.68 (0.46)

^a^ All correlation coefficients in parentheses were numbers reported by Huesken. ^b^ Both training and test sets were represented on the basis of 21-NT sequences.

## Data Availability

Not applicable.

## Supplementary Materials

The following supporting information can be downloaded at: https://www.mdpi.com/article/10.3390/molecules27196412/s1, the original siRNA sequences for Huesken and Bramsen datasets, the corresponding BCUT values and the data related to the partitions and predictions are provided in respective Supplemental files.
