# Supplementary material for: Cheminformatics Modeling of Gene Silencing for Both Natural and Chemically Modified siRNAs
Source: Molecules. 2022 Sep 28;27(19):6412. doi: 10.3390/molecules27196412 (PMC9570765; doi:10.3390/molecules27196412)
Supplement: Supplementary file 1 [file molecules-27-06412-s001.zip › BramsenDataSet/README(Bramsen).pdf]

<u>This folder contains data files related to the Bramsen dataset and its modeling</u>

SMILES structures and their ID used for all the chemically modified nucleotides used in the Bramsen paper -

Bramsen_modified_nts_struct_xl

Three pairs of training and test datasets (siRNA ID, potency and BCUT values) -

Bramsen_train4_forModel1 and Bramsen_test4,

Bramsen_train10_forModel2 and Bramsen_test10,

Bramsen_train22_forModel3 and Bramsen_test22

Three pairs of predicted and actual potency -

Bramsen_corr_plot_train_model_set04_04

Bramsen_corr_plot_test_model_set04_04;

Bramsen_corr_plot_train_model_set10_04

Bramsen_corr_plot_test_model_set10_04;

Bramsen_corr_plot_train_model_set22_04

Bramsen_corr_plot_test_model_set22_04.

BCUT values of all the chemically modified siRNAs used in the Bramsen paper -

Bramsen_xl_modified_nts_bcuts

Original IDs, potency (eGlogP), and siRNA sequences used in Bramsen paper -

modified_siRNA_AS_Bramsen_xl_dataset
