# Supplementary material for: Cheminformatics Modeling of Gene Silencing for Both Natural and Chemically Modified siRNAs
Source: Molecules. 2022 Sep 28;27(19):6412. doi: 10.3390/molecules27196412 (PMC9570765; doi:10.3390/molecules27196412)
Supplement: Supplementary file 1 [file molecules-27-06412-s001.zip › HueskenDataSet/README (Huesken).pdf]

<u>This folder contains data files related to the Huesken dataset and its modeling</u>

ACGUT_bcuts

- The calculated BCUT values for all five natural nucleotides

Huesken_ID-21ntSeq-NormAct_2431

- ID, potency and siRNA sequence

Huesken-train-test-values-9-27-2022

- ID, potency, 252 BCUT values and predicted potencies for the training set, shown in the last 2153 rows, and test set shown in the first 278 rows of this spreadsheet.

rational_split.cpp

- The CPP code for conducting splitting
